# Homeopathy and integrative medicine: keeping an open mind

**DOI:** 10.1007/s12682-014-0198-x

**Published:** 2014-12-17

**Authors:** Paolo Bellavite

**Affiliations:** Department of Pathology and Diagnostics, University of Verona, Verona, Italy

**Keywords:** Complementary medicine, Integrative medicine, Homeopathy, History of medicine, Physician—patient relationship, Evidence in medicine

## Abstract

Some physicians have incorporated some forms of complementary and alternative medicine (CAM) or related medicinal products in their clinical practices, suggesting that an unconventional treatment approach might be seen as an integration rather than as an alternative to standard medical practice. Among the various CAMs, homeopathy enjoys growing popularity with the lay population, but it is not acknowledged by academia or included in medical guidelines. The major problem is to establish the effectiveness of this clinical approach using the strict criteria of evidence-based medicine. This issue of the Journal of Medicine and the Person collects contributions from some of the most prestigious centers and research groups working in the field of homeopathy and integrative medicine. These contributions are not specialized information but are of general interest, focusing on this discipline as one of the emerging fields of personalized medical treatment.

Advances in biotechnology have led to novel diagnostic and therapeutic approaches that have rendered medicine both safer and more efficacious. In spite of this progress (or maybe because of it) the interest in alternative and non-conventional forms of medicine is also growing (for common definitions see Box 1 in “Appendix”). Patients intimidated by the complexity and the cost of biotechnology may find these therapies more friendly and more congruent with their personal desires. Complementary and alternative medicine (CAM) claims to take advantage of remedies present in nature and to minimize or refuse the use of synthetic medications. These are seen as foreign substances that threaten the wholesomeness of the human body, but these thoughts can have misleading consequences.

To be able to meet the expectations of their patients, more and more physicians are trying to understand—instead of discarding “a priori”—these alternative therapeutic methods [1]. Some physicians have even incorporated some forms of alternative medicine or products in their clinical practices. It is not rare to hear respected clinicians suggesting that an unconventional treatment approach might be seen as an “integration” rather than as an “alternative” to standard medical practice. In the opinion of these professionals the integration of conventional and unconventional medicine may lead to improved outcomes, improved patient satisfaction, and improved treatment cost/effectiveness. Oriental medical systems (Chinese medicine, ayurveda) and homeopathy, and anthroposophic medicine in the West were based on specific pathophysiological theories, semiotic methods, and pharmacopeias that may deserve re-evaluation. At the very least, the study of these methods may lead to improved communication with and trust from patients, who feel empowered in their care when the doctor examines their requests with an open mind.

Together with the unprecedented increase in scientific and technical knowledge of recent decades, there has been an increase in the prevalence of complex conditions, characterized by pluri-morbidity, and associated with the aging of the population [2]. This calls for an individualized treatment approach, something that modern medicine may be ill equipped and ill prepared to do. Randomized clinical trials of medical treatment are essential to provide evidence of the efficacy or lack thereof of a specific treatment. But the results of these trials may not pertain to the complex clinical situation of the individual patient. Currently, the majority of diseases are multifactorial processes that may not be manageable with a single intervention but require a multifaceted approach. Complex diseases, such as diabetes, schizophrenia, cancer, and atherosclerosis, may involve hundreds of genomic variants that interact with one another and with environmental factors. This very complexity shows the inadequacy of a reductionist approach, aiming at discovering and correcting one or few molecular defects using targeted drugs. Other well known problems are the adverse effects and drug resistance. To give just one example, imatinib has been invaluable in improving the prognosis of patients with chronic myelogenous leukemia, but the longer these patients live, thanks to the drug, the more likely they are to develop resistance to its effect [3].

Alexis Carrel, a prominent Nobel Prize laureate in 1912, was one of the first medical scientists to envision the basic problems of modern medicine. Few people know that he was also interested in alternative medical approaches and oversaw the publication of a book devoted to the problems raised by “médecines hérétiques”, where he stated “It must be admitted that the advances in medicine are far from having eliminated the disease. Rather than dying quickly from infections patients nowadays die, more slowly and more painfully, as a result of degenerative diseases such as all types of chronic diseases including cancer, diabetes, cardiac failure, chronic renal failure and neurodegenerative ailments. Medicine did not reduce human suffering as much as we have believed and hoped for. We have become aware that suffering derives not only from nocuous agents, such as bacteria and viruses, but it may be caused by subtler and poorly defined conditions, such as the fragility of our brains and other aging organs” [4].

Chronic and degenerative diseases are both an effect and a cause of increased health care cost, which by itself may limit access to care even in the most developed countries [5]. While all economists agree that the situation is unsustainable, solutions are not easy to envision. In addition to a more cost-effective utilization of current medical resources, the solutions may include a novel anthropological attitude of medicine, where more attention is given to lifestyle and treatments are individualized. Alternative and complementary medicine may be part of the solution [6] when critically examined and adopted in accordance with the criteria of informed freedom of therapy and evidence-based medicine.

Homeopathy enjoys growing popularity with the lay population, but it is viewed with skepticism by academia and is still excluded from medical guidelines. Originated at the end of the eighteenth century by ideas and experiments of Christian Friedrich Samuel Hahnemann (1755–1843), homeopathy is the only Western medical system that “survived” the advances of modern medicine. The delayed recognition of the possible contribution of homeopathic ideas to mainstream medical science and, on the other hand, the uncritical acceptance and insistent attacks by some homeopaths against allopathy are at least partially responsible for the rejection of homeopathy by the majority of modern physicians and academic circles. Since its inception homeopathy has presented a twofold nature. One is a holistic approach aimed at treating the individual as a whole (individualized treatment); the other is a data-driven approach abiding by experimental methods. Current medical literature is “opening up” to homeopathy, as documented by the appearance of several journals dedicated to the field and their inclusion in the main databases. For example, the number of the papers that deal with homeopathy cited in PubMed is currently (October 2014) 5,538, while in 2000 there was less than one-third of this number [7]. Contrary to what is gratuitously believed, most of the traditional concepts proposed by homeopathy (the principle of “similarity”, drug experimentation on healthy people, the individualization of prescription, the use of very low doses of medicines) are germane to scientific criteria [8]. The major problem homeopaths encounter is to establish the effectiveness of their care using strict criteria of the statistical evidence and the double-blind trial; see also the paper of Viganò et al. [9] in this issue.

The principle of similarity holds that a “pathogenic” substance administered in small doses may correct the physiologic imbalance of a diseased organism presenting symptoms similar to those that the substance causes when tested in healthy people. This process is comparable to desensitization of allergic people with small doses of allergens. Likewise β-blockers that decrease the contractility of the normal heart may improve it in the presence of heart failure (paradoxical pharmacology). The antidepressants that may relieve melancholy in a depressed individual may cause it in a normal subject. These effects are only apparently paradoxical [8]. The homeostasis of any complex system, including the immunologic, cardiovascular, and nervous systems is based on the equilibrium of antagonistic activities of different substances or different receptors for the same substance. This homeostasis may become chronically disrupted in a situation named “dynamic pathologic attractor.” To reverse this condition and bring homeostasis back to the system, it may be necessary to trigger an endogenous therapeutic reaction, with a specific pathogenic substance contained in small doses in the homeopathic remedy.

The doses of most homeopathic drugs are small but measurable. Though these drugs may be administered at very high dilution (even higher than the Avogadro’s constant that corresponds to the 12th centesimal dilution), they are different from any form of placebo. The water that is generally used as a diluent appears to have a mesoscopic structure. That means that the H_2_O molecules and other solutes form aggregates of millions of molecules (clusters or nanoparticles) may incorporate information from active substances. This mechanism may be analogous to the memory of microchips in flash drives.

This issue of the Journal of Medicine and the Person collects contributions from some of the most prestigious centers and research groups working in the field of homeopathy and integrative medicine. This is the first time that a non-homeopathic Journal has “opened the door” to this controversial topic in a special issue, and this is a credit to the courage and foresight of the publisher. As co-editor of this issue, I paid particular attention to ensuring that the contributions were not specialized information but of general interest: in fact these papers focus on this discipline as one of the emerging fields of personalized medical treatment.

The work of Viganò et al. [9] introduces the historical and philosophical bases of homeopathy together with its scientific fundaments. The article emphasizes that homeopathy originated as an experimental discipline rather than spawning from a series of theoretical concepts. Indeed it represented the first attempt to understand the effects of drugs through systematic experimentation on healthy subjects (“proving” of medicines). The disease must be studied as a whole (and not only in terms of its main symptom or pathology) to ensure that the disease and the drug interact in a global manner; the choice of the remedy must be based on the complex of individual symptoms rather than on the name of the disease. The article explores the economic, historical, and conceptual barriers that have so far prevented the acceptance of homeopathy by mainstream medicine. Homeopaths are “forced” to work in a conceptual and operational system not recognized in the academic environment. The future will tell us whether the effort to “prove” homeopathy according to the qualitative and quantitative criteria accepted as scientific methods (pre-clinical studies, clinical trials, epidemiological studies) will be successful.

Then Bonamin and Waisse [10] from the University of São Paulo describe their original perspective for interpretation of homeopathy. Their work tends to challenge the common opinion according to which homeopathy is unscientific precisely because homeopathic medicines—when diluted beyond Avogadro–Loschmidt constant—have no matter whatsoever. They present the position of “biosemiotics” that the images “significant” for living beings—including drugs—are not immediate, but “mediated” through signs. Signs might be chemical, electric, magnetic, thermal, acoustic or mechanical. Signs of biologic interest are also the frequencies at which some phenomena occur, as is the case in neuronal transmission. Homeopathic medicines then involve a material vehicle (the grains, drops, tablets, etc.) and the “sign” of the original drug principles. Interestingly, these models tend to infer how the “signs” introduced by the homeopathic treatment may touch sensitive systems in the physiology and pathology of the patient, thereby boosting and directing the healing process. Finally, as suggested by Waisse and Bonamin, these views do not apply to homeopathy only, but become an endless source for studies aiming to achieve a more refined understanding of living beings and their relationships with the environment.

Disease and healing have both subjective and objective dimensions, which may or may not co-exist. Observational studies assessing “quality of life” changes during long-term homeopathic therapy showed beneficial effects even in the absence of significant improvements of laboratory or electro-physiologic parameters [11]. The paper by Koithan et al. [12] describes the individual experiences of homeopathic patients that lead to a better understanding of the patient perspective in the therapeutic process. Their results indicate how the patient’s phenomenological experience of healing during homeopathic treatment involves a transformational experience entailing, among other things, the patient–provider relationship. A trusted partner in care facilitates self-exploration and self-discovery. Interestingly, patients report that the treatment helped them to become “unstuck” from chronic dysfunctional patterns at the somatic, mental, social, spiritual, and developmental levels. This study confirms the claim that homeopathy triggers a global healing response resulting in a greater sense of “freedom” at multiple levels.

The risks inherent in healing practices with no scientific bases should not be underestimated. These include wasting of resources, delay or avoidance of effective therapy, and the widespread prejudice against treatment based on scientific evidence. This prejudice involves a dualistic and Manichean vision of medicine: what comes directly from nature is good and what is produced through biotechnology is wicked. Furthermore, some Oriental medical and parapsychological practices with the most varied and imaginative applications may be used to promote philosophical systems, ways of thinking, ways of life, or even esoteric beliefs. In our opinion, this approach does a disservice to both medicine (understood as the prevention and cure of disease) philosophy (understood as an attempt to explain reality through reasoning) and religion (understood as personal and social experience of encounters with the divine). The relationship between medicine, philosophy, and spiritual experience is a difficult and delicate issue, with the opposite risks of overstating and of overlooking the healing potential of different human dimensions. The paper by Swayne [13] illustrates how this issue may be examined with a rational approach. Medicine may be enriched and rendered more effective with spiritual beliefs and experiences. Clearly this is not pertinent to homeopathy only, and the author presents homeopathy, a discipline that he is practising as physician, as just one example of this complementarity.

The last three contributions illustrate the situation of homeopathy and integrative care in three different geographic areas. Rossi et al. [14] describe the integration of homeopathy into the national health system in Tuscany, one of the most developed Italian regions. Tuscany has been a “laboratory” of integrative medicine for many years and now it is conducting a pilot project that may help future scientific developments and regulatory acceptance of homeopathy. Thanks to the commitment of several physicians and of the Italian Society of Homeopathy and Integrated Medicine, CAMs are also utilized in surgeries belonging to the public health system and even in the Hospital of Pitigliano (Grosseto). It is the duty of the government to submit any promising form of medical treatment to tests of efficacy and risk and this may include homeopathic treatment whose value should be demonstrated in the proper scientific context. The pilot experience of the Tuscany region in Italy, as reported by Rossi and co-workers in this issue, is particularly illuminating and useful as a preliminary application of these principles.

Quirk and Sherr [15] are two doctors who established a well-structured homeopathic practice in Tanzania, East Africa. They describe how the working group “Homeopathy for Health in Africa” offers patients an integrative, holistic method to supplement standard medical treatment and mitigate the side-effects of anti-retroviral (ARV) drugs that often interfere with patient adherence to treatment and lead to drug resistance. Cases are presented that show how patients who have homeopathic treatment as a supplement to ARVs report amelioration of side-effect symptoms, increased energy, and enhanced well-being, allowing them to work and care for their families. Clearly, this report from Africa represents an encouraging example of integrative health care more than a study of drug efficacy.

Ben-Arye and Samuels [16] from Lin Medical Center, Haifa, Israel, examine the role of homeopathy in the context of an evolving acceptance of complementary medical practices among Middle-Eastern medical practitioners, as well as within the framework of clinical practice. The Middle East is characterized by a rich spectrum of complementary and traditional therapies, which are used by patients in parallel with conventional medicine. It was interesting to compare the foundations of homeopathy with those of traditional Middle-Eastern medicine and explore the possibilities of collaborative research and clinical practice in Middle-Eastern health systems.

Faced with the challenges posed by the spread of “alternative” medical practices, official medicine can no longer ignore the phenomenon; it needs to adapt its methods and to pose the question of the possible integration of different therapeutic settings in a pluralistic health care system with appropriate safeguards of efficacy and safety. A possible integration path does not include the assertion of the superiority of one method or another, but the consideration that in the complexity of many diseases no treatment method may have exclusivity. Integrative medicine is not the “merging” of alternative medicine with conventional biomedicine. It represents a higher-order system of care that emphasizes wellness and healing of the entire person (bio-psycho-socio-spiritual dimensions) as primary goals, drawing on different medical approaches in the context of a supportive and effective physician—patient relationship [17]. This approach is particularly effective in the supportive care in oncology [18–20], geriatrics [21], pediatrics [22] neurology [23–25], fibromyalgia [26], and even for the physicians’ health and wellness [27].

As with all therapies, complementary ones have their contraindications, which operators need to know as well as the potential indications. Without going into the details of each individual subject, it is appropriate to point out a problem common to all complementary medicines: the risk that the patient be “diagnosed” and treated with unconventional methods which ignore the diagnosis so that some diseases, even serious ones, can go unnoticed. Another risk is that patients and doctors themselves are not able to judge objectively the outcome of care, in the absence of instrumental parameters and laboratory reports. This could be ameliorated by a more effective collaboration between unconventional therapist and conventional reference centers for follow-up therapies (e.g. antidiabetic centers, allergy, cardiovascular, mental health, and so on). Another risk derives from the fact that herbal preparations from Eastern countries are subjected to fewer and looser controls than conventional drugs before being placed on the market. They may be contaminated with active ingredients other than those stated or have expired. Caution in the field of therapies not fully consolidated is a must, but should not prevent the exploration of the potential benefits of these methods.

Early attempts to transform homeopathy from an empirical discipline to a scientific one, carried out especially in Germany and United States in the nineteenth century, are carefully described in a seminal book dated 1936 by Linn John Boyd, Professor of Medicine at the New York Homeopathic Medical College [28]. There we read that one of the greatest physicians in Germany at the time of Hahnemann was Christof Wilhelm Hufeland (1762–1836) a pioneer of medical journalism, editor of *Journal der Praktischen Arzneikunde*. Although he was a leading representative of official medicine, his works included many references indicating his openness to homeopathic ideas and his journal published several of Hahnemann’s papers. I want to end these introductory remarks with his thought-provoking quotation: “Prove all and hold fast to the good is and r*e*mains the first commandment of science. Medicine is a science of experience, practices a continuous experiment, and the experiment is not concluded. Freedom of thinking, freedom of science, that is our highest palladium and it must so remain if we are in progress. No type of despotism, no sole ruling, no suppression of thought. Even the government should not be permitted to invade scientific subjects, nor depress, or favor one opinion exclusively; both have, as experience teaches, done damage to the truth. Only proving through experience, discussion and counter-discussion, continuous free study, and time can and will surely in the end separate truth from falsity, the useful from the useless” (Hufeland, System der Praktischen Heilkunde, 1830, cited in [28]).

## Box 1: Definitions

### Complementary medicine (CM) or complementary and alternative medicine (CAM)

The terms “complementary medicine” or “alternative medicine” refer to a broad set of health care practices that are not part of that country’s own tradition or conventional medicine and are not fully integrated into the dominant health-care system. According to WHO they are used interchangeably with “traditional medicine” in some countries. Homeopathic medicine, herbal medicine and acupuncture are the most common, albeit not the unique, CAM forms in European countries.

### Homeopathy

Homeopathy is a method of medical practice that aims to improve the level of health of an organism through the administration of medicinal products selected individually according to the principle of similarity (see text). Since homeopathy is strictly individualized and takes into account the physical, emotional, mental, constitutional, biographical and environmental state, it is a medicine of the whole person. The term homeopathy comes from the Greek (omoios = similar, pathos = suffering).

### Integrative medicine

Integrative medicine is not simply the combination of conventional medicine with complementary and alternative medicine. The Consortium of Academic Health Centers for Integrative Medicine defines it as “the practice of medicine that reaffirms the importance of the relationship between practitioner and patient, focuses on the whole person, is informed by evidence, and makes use of all appropriate therapeutic approaches, healthcare professionals and disciplines to achieve optimal health and healing”.
